# Quality of life and complications in elderly patients after pronation rotation type III ankle fractures treated with a cast and early weight-bearing

**DOI:** 10.1186/s12891-021-04745-0

**Published:** 2021-10-14

**Authors:** Alejandro Lorente, Antonio Gandía, Gonzalo Mariscal, Pablo Palacios, Rafael Lorente

**Affiliations:** 1 Department of Traumatology and Orthopaedic Surgery, University Hospital Ramón y Cajal, M-607, km. 9, 100, 28034 Madrid, Spain; 2 Institute for Research on Musculoskeletal Disorders, School of Medicine, Valencia Catholic University, 46001 Valencia, Spain; 3 Department of Traumatology and Orthopaedic Surgery, University Hospital Madrid Sanchinarro, Calle de Oña, 10, 28050 Madrid, Spain; 4 Department of Orthopedic Surgery and Traumatology, University Hospital of Badajoz, Av. de Elvas, s/n, 06080 Badajoz, Spain

**Keywords:** Ankle fracture, Pronation external rotation, Weight-bearing, Elderly patients, Quality of life

## Abstract

**Background:**

Early weight-bearing is becoming increasingly common because it can positively affect the quality of life of patients. Therefore, the efficacy and safety of this conservative treatment should be assessed for different types of ankle fractures. The goal of this study was to compare early weight-bearing and non-weight-bearing in terms of effectiveness and safety in patients with pronation rotation type III ankle fractures treated nonsurgically.

**Methods:**

A prospective multicenter cohort study was conducted over two years. Elderly patients with a nondisplaced pronation rotation type III ankle fracture were included. The main variables were the Barthel Index and SF-12 scores. The patients completed the questionnaires at six weeks, one year and two years. We also compared the complications associated with the two interventions.

**Results:**

30 patients were included in the weight-bearing group, while 32 patients were included in the non-weight-bearing (WB) group. The mean ages were 82.6 ± 2.6 years and 83.1 ± 2.6 years, respectively. Quality of life, measured with the SF-12 scale, increased significantly in both the short and long term in the WB group (53.5 ± 5.8 points vs 65.2 ± 4.4 points at 6 weeks and 70.1 ± 4.2 points vs. 80.9 ± 3.7 points at 2 years; *p*<0.001). The WB group also showed a higher quality of life, as measured by the Barthel Index (54.5 ± 5.2 points vs. 64.3 ± 4.0 points at 6 weeks and 71.0 ± 4.3 points vs. 80.7 ± 3.4 points at 2 years; *p*<0.001).

**Conclusions:**

Elderly patients with pronation rotation type III fractures could benefit from an early weight-bearing protocol in terms of quality of life and functionality.

## Background

Pronation rotation type III fractures account for approximately 10.6% of all ankle fractures. However, surgeries for this type of fracture account for nearly 40% of all ankle surgeries [1]. The controlled use of compression and controlled loading enhances bone healing, even the application of controlled shearing and distraction are useful for fracture healing [2].

The stability of the fracture is a crucial factor for surgical or conservative management [3]. Some studies suggest that surgical treatment yields better radiological outcomes than the conservative approach [4]. Regarding displaced fractures, open reduction and internal fixation yield better function and a larger range of motion than nonoperative treatment [4].

Although the indications for surgical intervention for ankle fractures are theoretically well defined, the rate of surgery varies widely in the current literature. The decision of whether to perform surgery is based on factors such as sex, age, and comorbidities (diabetes or peripheral vascular disease) [5].

However, most of these previous studies were conducted in younger patients [6]. In elderly patients with nondisplaced weber type C fractures with deltoid ligament injuries (or the equivalent nondisplaced medial malleolar fractures), conservative treatment can be chosen, given the risk of surgery-related comorbidities [7]. In addition, age may negatively influence functional outcomes after surgery [8].

Whether to implement early weight-bearing (WB) (this means loading the limb properly using a plastic brace or crutches) or non-weight-bearing (NWB) remains controversial. More randomized studies are needed since most of the existing studies are cohort studies [9]. Conventionally, non-weight-bearing for 6–8 weeks after surgery is advised [10]. There is still no consensus on the optimal NWB or WB time. Some of the factors that led the orthopedist to extend the NWB time were bone quality, presence of comorbidity, advanced age and disability [11].

Favorable radiological results have been demonstrated in patients older than 80 years who adequately performed non-weight-bearing for 6–8 weeks after surgery [12]. Immobilization in a cast may result in adverse events such as ankle stiffness, impaired ankle range of motion and delayed return to work [13].

Controlled weight-bearing, that is, axial compression forces, can be beneficial for rehabilitation outcomes and quality of life. The physiological and biomechanical roles of controlled weight-bearing are well known [14–16]. Early mobilization can accelerate recovery and improve mobility and quality of life. However many of these studies are performed after surgery (ORIF) [10].

For other types of fractures, such as bimalleolar fractures, early weight-bearing increases quality of life scores more than non-weight bearing does. Non-weight-bearing patients remain in a wheelchair or bedridden for a long time [17].

This study focuses on pronation rotation type III fractures. The goal of this study was to compare early weight-bearing and non-weight-bearing in terms of effectiveness and safety in patients with pronation rotation type III ankle fractures treated nonsurgically.

## Methods

### Design

A multicenter prospective cohort study was conducted from January 2015 to December 2018. Sixty-two patients provided their consent to participate in the study. The study was approved by our institution’s ethics committee and the entire study adhered to the Declaration of Helsinki guidelines for research in humans. The manuscript fulfil all the relevant reporting standards for the cohort study (STROBE) [18].

### Participants

**Fig. 1:**
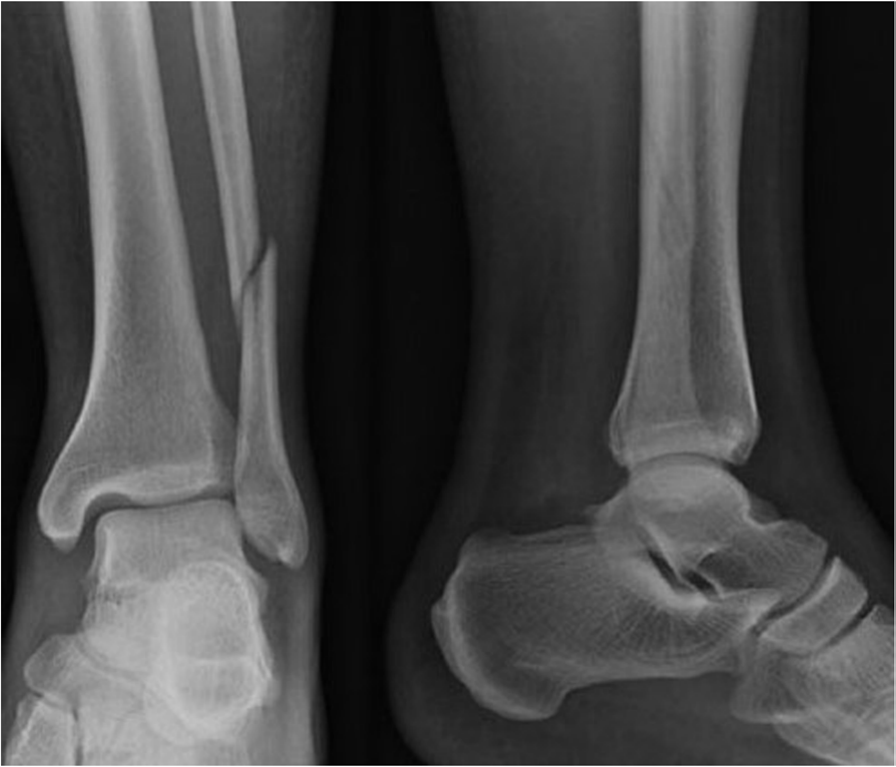
A non-displaced pronation rotation type III akle fracture with deltoid ligament injury, anterior syndesmosis (anterior tibiofibular ligament) injury, and non-displaced medial malleolus

The inclusion criteria were patients over 80 years old with a type III rotational pronation fracture [19, 20], i.e., a nondisplaced Weber type C fibular fracture (less than 2 mm displacement), a deltoid ligament injury, anterior syndesmosis injury (anterior tibiofibular ligament), fracture of the distal fibula and a nondisplaced medial malleolus (Fig. 1). Fractures were classified by and orthopaedic surgeon and a radiologist. If there were any doubts or discrepancy between them, a third doctor (another orthopaedic surgeon) was consulted. Patients were included consecutively. The exclusion criteria were patients with an injury of the posterior malleolus or a pronation type IV external rotation fracture, patients admitted for a different reason, and patients in whom the cast was broken [17]. Two patients were excluded from the WB group for weight-bearing [17]. Incorrect weight-bearing was defined as the inability of the patient to completely step the foot on the ground. This fact was verified by the orthopaedic surgeon during the follow up period in the clinical room. Some patients avoided such thing due to pain or discomfort. If those patients were not-loading “completely” it would have been a bias to include them in the weight bearing group. Three were excluded from the NWB group for death from infectious pneumonia (one patient) and prolonged internal medicine admission (two patients). Patients with prolonged internal medicine admission) were excluded since it could bias the SF 12 outcomes. This methodology was performed according to previous studies and to avoid bias [17].

### Data collection

The baseline characteristics of each patient were retrieved from hospital records. The main variables consisted of scores reflecting functionality and quality of life. The scales used consisted of the SF-12 index, which measures quality of life in relation to physical and mental health. It consists of 12 items addressing the 8 dimensions of the SF-36: physical function, social function, physical role, emotional role, mental health, vitality, body pain and general health. Studies that used the SF-12 index have confirmed that this is a valid and reliable assessment tool [21]. The Barthel Index was also assessed. The Barthel Index measures the capacity of a person to execute ten basic activities in daily life, leading to a quantitative estimation of the subject’s level of dependency, it is commonly used to measure quality of life after a lower limb fracture, specially proximal femur ones, but it is also used to measure quality of life after other types of lower limb fractures including the ankle [17, 22–25]. The rage of these two questionnaires is 0 (the worst health status for that dimension) to 100 (the best health status). These assessment tools were administered at 6–8 weeks and at one and two years. Questionnaires were collected in person by the orthopaedic surgeon during the follow up period in the clinical room. We used an electronic database.

The complications assessed included secondary displacement of the fracture, non-union of the medial and lateral malleolus (lack of bone healing after 6 months), soft tissue injuries, infection, implant failure and rescue surgical intervention. Complications were verified by the orthopaedic surgeon during the follow up period in the clinical room. A displaced fracture was defined as a fracture with more than 2 mm of displacement regarding both the fibula and medial malleolus. Ankle mortise was considered to be dislocated (displaced) if there was < 6 mm tibiofibular overlap in the AP view and < 1 mm on the mortise view. Infections, implant failures and complications in general were verified by the orthopaedic surgeon during the follow up period in the clinical room.

### Intervention

The patients were first admitted to the emergency department, and patients with associated injuries were excluded. Patients meeting inclusion criteria were placed in a closed cast, and nonsurgical intervention was indicated. The first follow-up was performed 7 days afterwards. AP, lateral and mortise radiographs were then performed again. Patients in whom the fracture was not displaced were included in the study. Once the absence of displacement of the fracture was verified, the patients assigned to the weight-bearing or non-weight-bearing group. To ensure a homogeneous distribution of the sample and avoid bias, the inclusion of the patient in the case or control group was chosen depending on whether the day of the first visit to the clinic was odd or even. The patient was treated firstly in the emergency department and afterwards was followed up in 7 days in Clinic. If the day of the first clinic was even (for example 16th of April), the patient was automatically assigned to the early weight-bearing group while if the day was odd, the non-early-weight-bearing group was assigned.

Patients belonging to the weight-bearing group were taught how to load their limb adequately using a plastic brace or crutches. Correct loading was defined as ability of the patient to completely step the foot on the ground. This fact was verified by the orthopedic surgeon during the follow up period in the clinical room. To exclude secondary displacements and ensure adequate fracture healing, control X-rays were performed every 2 weeks until the sixth or eight week. Both groups used the same type of plaster cast, which was maintained for 6–8 weeks, depending on the extent of consolidation viewed on the follow-up X-rays.

### Statistical analysis

The categorical descriptive data are shown as numbers and percentages. The continuous variables are shown as means and standard deviations. The 95% CIs were also calculated. Independent 2-tailed sample t-tests were used to compare the main outcomes between the two groups. The relationship between complications and the type of load, two nonquantitative variables, was assessed through a chi-square test and *p* < 0.05 was considered statistically significant. The statistical analysis was carried out using the SPSS package, v. 24.0 (IBM, USA).

## Results

### Baseline data

A total of 67 patients were enrolled at the beginning of the study. Table 1 shows the baseline features and mean SF-12 and Barthel’s Index scores measured at 6 weeks, one year, and two years. A total of 62 patients were enrolled: 32 and 30 patients were included in the NWB and WB groups, respectively. The average age was 82.6 ± 2.6 in the NWB group and 83.1 ± 2.6 in the WB group. For sex, the proportions of males were 40.6 and 50.0% in the NWB and WB groups, respectively.

**Table 1:** Baseline data and quality of life evaluated by SF-12 and Barthel Index

	Mean Age	% Male	SF-12 Initial	6 weeks	1 year	2 year	Barthel Initial	6 weeks	1 year	2 year
NWB (*n* = 32)	82.6 ± 2.6	40.6%	84.1 ± 3.6	53.5 ± 5.8	65.2 ± 5.1	70.1 ± 4.2	85.4 ± 3.4	54.5 ± 5.2	66.6 ± 4.6	71.0 ± 4.3
WB (*n* = 30)	83.1 ± 2.6	50.0%	85.4 ± 3.8	65.2 ± 4.4	78.7 ± 3.5	80.9 ± 3.7	86.0 ± 3.6	64.3 ± 4.0	79.1 ± 3.8	80.7 ± 3.4

*Abbreviations*: *WB* weight-bearing, *NWB* non-weight-bearing

### Clinical variables

**Fig. 2:**
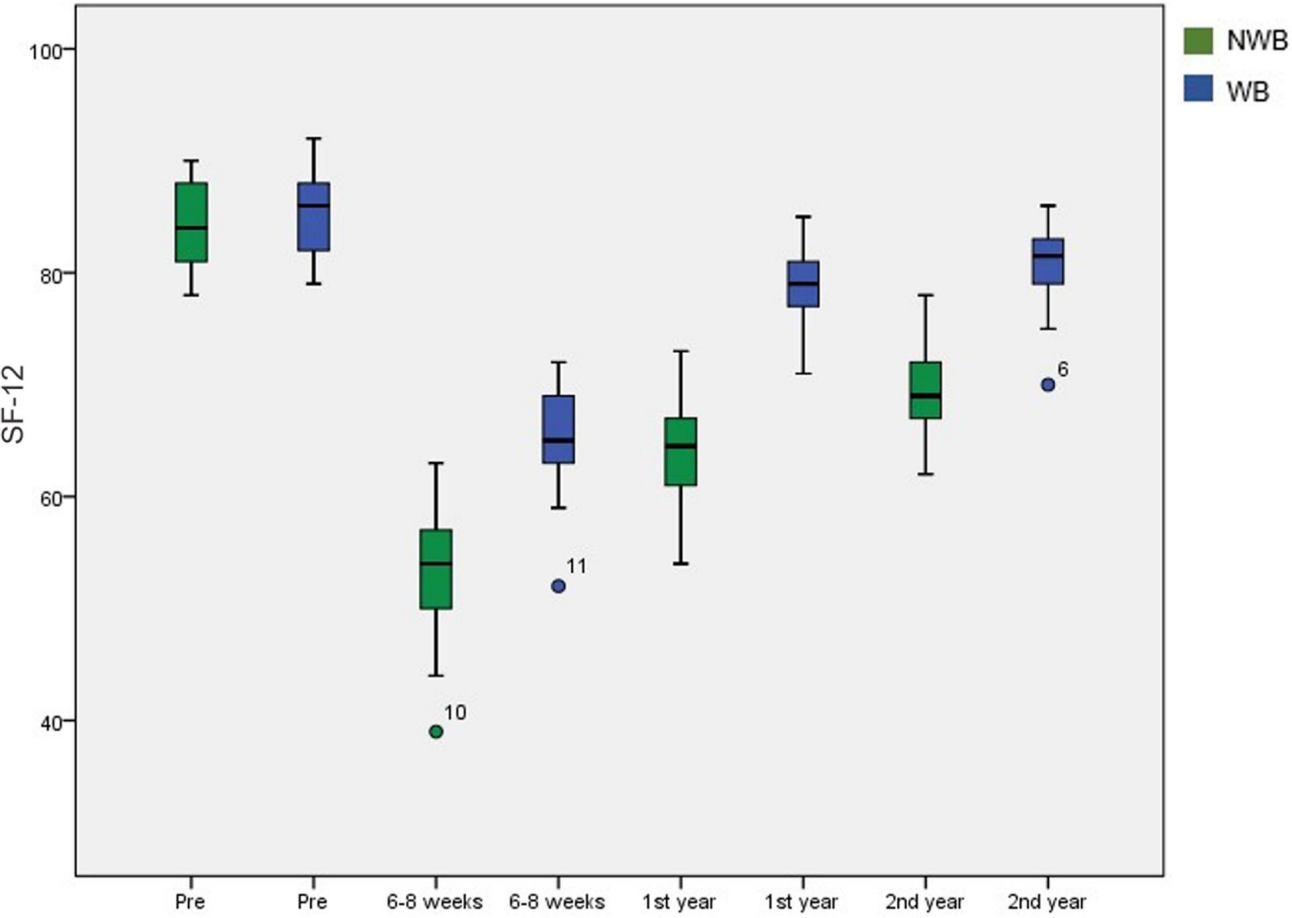
Box plot of the progress of the quality of life measured through the SF-12 scale. Patients in the WB group showed a significantly higher score during follow-up

**Fig. 3:**
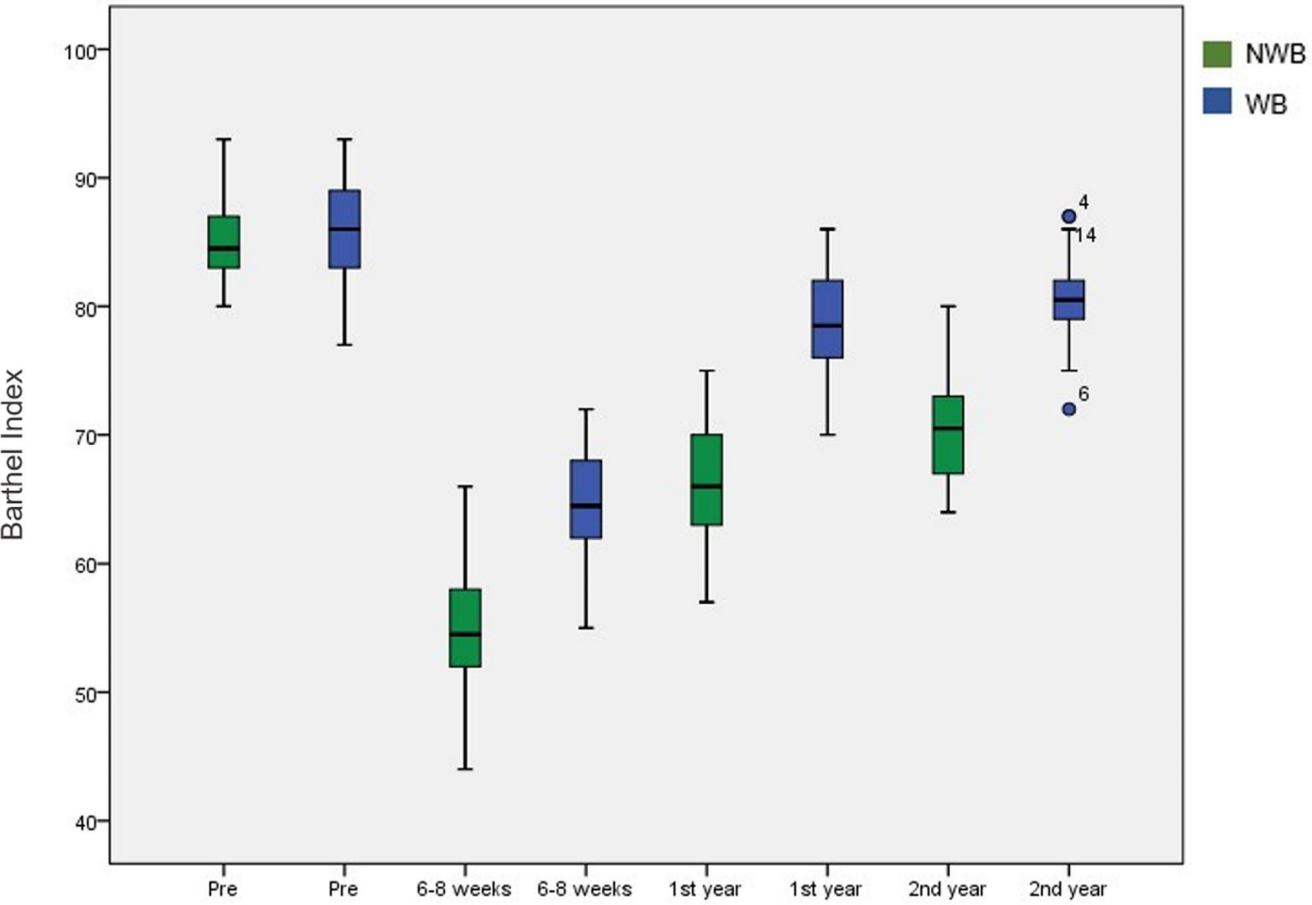
Box plot of the progress of the quality of life measured through the Barthel Index. Patients in the WB group showed a significantly higher score during follow-up

The initial SF-12 score was 84.1 ± 3.6 in the NWB group and 85.4 ± 3.8 (*p* > 0.05) in the WB group. At 6 weeks, the SF-12 score was 53.5 ± 5.8 in the NWB group and 65.2 ± 4.4 in the WB group (*p* < 0.001). After one year, quality of life improved, and significant differences were found between the two groups (65.2 ± 5.1 vs. 78.7 ± 3.5; *p* < 0.001). Finally, after two years, quality of life was higher in the WB group (70.1 ± 4.2 vs. 80.9 ± 3.7; *p* < 0.001) (Fig. 2). Initially, the Barthel Index, which reflected quality of life, was 85.4 ± 3.4 in the NWB group and 86.0 ± 3.6 in the WB group. Quality of life at 6–8 weeks showed significant differences in favor of the WB group (54.5 ± 5.2 vs. 64.3 ± 4.0; *p* < 0.001). There were significant differences at one year in favor of the WB group (66.6 ± 4.6 vs. 79.1 ± 3.8; *p* < 0.001). At 2 years, quality of life was higher in the WB group than in the NWB group (71.0 ± 4.3 vs. 80.7 ± 3.4; *p* < 0.001) (Fig. 3).

### Adverse events

We found no differences in complications between the two groups in the categories of complications. There were 18 (56.3%) adverse events in the NWB group and 18 (60%) in the WB group. There were no significant differences between the two groups. In the NWB group, 3 patients presented secondary fracture displacement, 3 non-unions of the medial malleolus, 2 non-unions of the lateral malleolus, 7 soft tissue problems and 3 rescue surgeries. In the WB group, 4 patients presented secondary fracture displacement, 3 non-unions of the medial malleolus, 2 non-unions of the lateral malleolus, 5 soft tissue problems and 4 rescue surgeries. There were no significant differences in terms of the different types of complications between the two groups (Table 2).

**Table 2:** Complications

Group	Non-weight-bearing	Weight-bearing	*p* - value
Secondary fracture displacement	3	4	*p* > 0.05
Non-union (medial malleolus)	3	3	*p* > 0.05
Non-union (lateral malleolus)	2	2	*p* > 0.05
Soft tissue problems	7	5	*p* > 0.05
Rescue Surgery	3	4	*p* > 0.05

## Discussion

The traditional approach of non-weight-bearing has been debated by different authors. The advantages and disadvantages of each procedure in the short and long term have been reported [15]. The application of early weight-bearing in stable fractures without soft tissue involvement and with reduced syndesmosis has also been described [26, 27].

However, it is important to consider the objectives of each procedure as well as the age and comorbidities of each patient individually.

In this study, elderly patients who underwent early weight-bearing from the beginning demonstrated an increase in their quality of life and functionality, as measured through the Barthel Index and SF-12 scale [17]. These two assessment tools are simple and reliable methods for assessing quality of life [28]. The quality of life questionnaires showed significant differences, but are these differences clinically relevant? There is no literature to answer this point in the case of ankle fracture. However, from the Barthel index ordinal variables are obtained according to the score range (e.g. between 50 and 74 means moderate dependence and between 74 and 90 means mild dependence). In our study the patients had a mean quality of life of 71 points in the NWB group and 81 points in the WB group at the end of follow-up. Thereby, in our study placing patients on WB means moving from moderate to mild dependence. Clinical variables such as pain influence the prognosis of ankle fractures. Patients with more pain will have larger limitations in the short and medium term [29].

The patients showed improvement in the short and long term, with the quality of life in the weight-bearing group being almost the same as that before the fracture. Moreover, these results are consistent with those in other studies, such as a study by Smeeing et al. This meta-analysis suggested that early weight bearing decreases the time to return to work and increases quality of life [15]. Early weight-bearing has yielded favorable results in other types of ankle fractures, such as bimalleolar fractures [17]. Other studies applied physical therapy after ankle fractures. Applying controlled physical activity yields good clinical results in 66% of patients [30, 31].

Our study is on the conservative management of fractures. We included patients over 80 years old. This is the age group that is considered orthogeriatric. Treatment without weight-bearing may have worse consequences in this population than in a young patient population. This is due to the changes produced by aging, such as worse fracture healing or the inclination to develop fibrous tissue.

However, the true adverse events of this procedure are reflected in quality of life scores. For example, weight-bearing can increase the rate of fracture displacement or non-union. Early weight-bearing is a process that can facilitate recovery in patients [14, 32].

Nonactivity or misuse as a result of immobilization, bed rest, a lack of loading or denervation causes numerous adaptive changes in the muscle to degenerate into muscle dysfunction as a final result. The loss of muscle mass or muscle atrophy is one of the consequences of muscle misuse. Several human studies have shown a loss of 30% of muscle mass during some weeks of disuse [33–35].

Few studies have evaluated the reversibility of disuse-induced muscle changes. There are studies that evaluated the effects of disuse. On the other hand, there are studies that combined immobilization with electrical evoked contractions during and after immobilization, resulting in a better muscle prognosis [36].

This study included a homogeneous sample of patients in terms of baseline characteristics (age, sex). Additionally, the two groups of patients at the beginning of the study showed a similar quality of life (Barthel and SF 12 scores) prior to the trauma. Thus, the baseline values were also homogeneous.

Several authors have argued that other possible risk factors related to the development of complications in patients with fractures, such as BMI, diabetes, tobacco use, and corticosteroids, should be documented since they are associated with a higher rate of complications in patients with some ankle fractures [37–43].

One of the limitations of the study was that other possible risk factors related to the development of complications in patients with fractures, such as BMI, COPD, diabetes, tobacco consumption and the use of corticosteroids, were not recorded. A larger sample size would also be required for the assessment of adverse events. The frequencies observed in each of the groups were low. Also, patients were followed by different orthopedic specialists. Related to the quality of life measure, there is a lack of evidence for the use of this measurement in the specific patient population, therefore it’s validity and reliability are unknown.

More research focused on patient compliance with weight-bearing recommendations and prospective and randomized studies should be conducted to help surgeons make more accurate recommendations for their patients about optimal weight-bearing after pronation rotation type III ankle fractures ankle fractures.

## Conclusions

In conclusion, in patients with pronation rotation type III fractures who are nonsurgical treatment candidates, early weight-bearing can increase their quality of life and functionality, and early mobilization enhances patient autonomy. Early loading is a promising and safe procedure with a low complication rate.

## Data Availability

Not applicable.

## Abbreviations

WB: Weight Bearing
NWB: Non-Weight Bearing

## References

[1] Juto H, Nilsson H, Morberg P (2018). Epidemiology of adult ankle fractures: 1756 cases identified in Norrbotten County during 2009-2013 and classified according to AO/OTA. BMC Musculoskelet Disord.

[2] Baker CE, Moore-Lotridge SN, Hysong AA, Posey SL, Robinette JP, Blum DM (2018). Bone fracture acute phase response-a unifying theory of fracture repair: clinical and scientific implications. Clin Rev Bone Miner Metab.

[3] Nortunen S, Lepojärvi S, Savola O, Niinimäki J, Ohtonen P, Flinkkilä T (2014). Stability assessment of the ankle mortise in supination-external rotation-type ankle fractures: lack of additional diagnostic value of MRI. J Bone Joint Surg Am.

[4] Hughes JL, Weber H, Willenegger H, Kuner EH (1979). Evaluation of ankle fractures: non-operative and operative treatment. Clin Orthop.

[5] Cheung Y, Perrich KD, Gui J, Koval KJ, Goodwin DW (2009). MRI of isolated distal fibular fractures with widened medial clear space on stressed radiographs: which ligaments are interrupted?. AJR Am J Roentgenol.

[6] Makwana NK, Bhowal B, Harper WM, Hui AW (2001). Conservative versus operative treatment for displaced ankle fractures in patients over 55 years of age. A prospective, randomized study. J Bone Joint Surg (Br).

[7] Keene DJ, Lamb SE, Mistry D, Tutton E, Lall R, Handley R (2018). Three-year follow- up of a trial of close contact casting vs surgery for initial treatment of unstable ankle fractures in older adults. JAMA.

[8] Hohmann E, Foottit F, Tetsworth K (2017). Relationships between radiographic pre- and postoperative alignment and patient perceived outcomes following Weber B and C ankle fractures. Foot Ankle Int.

[9] Phillips WA, Spiegel PG (1979). Editorial comment: evaluation of ankle fractures: non-operative versus operated. Clin Orthop.

[10] Thomas G, Whalley H, Modi C (2009). Early mobilization of opera-tively fixed ankle fractures: a systematic review. Foot Ankle Int.

[11] Swart E, Bezhani H, Greisberg J (2015). Vosseller JT (2015) how long should patients be kept non-weight bearing after ankle fracture fixation? A survey of OTA and AOFAS members. Injury.

[12] Fong W, Acevedo JI, Stone RG, Mizel MS (2007). The treatment of unstable ankle fractures in patients over eighty years of age. Foot Ankle Int.

[13] Schepers T, De Vries MR, Van Lieshout EM, Van der Elst M (2013). The timing of ankle fracture surgery and the effect on infectious com-plications; a case series and systematic review of the literature. Int Orthop.

[14] Vandenborne K, Elliott MA, Walter GA, Abdus S, Okereke E, Shaffer M (1998). Longitudinal study of skeletal muscle adaptations during immobilization and rehabilitation. Muscle Nerve.

[15] Smeeing DP, Houwert RM, Briet JP, Kelder JC, Segers MJ, Verleisdonk EJ (2015). Weight-bearing and mobilization in the postoperative care of ankle fractures: a systematic review and meta-analysis of randomized controlled trials and cohort studies. PLoS One.

[16] Tan EW, Sirisreetreerux N, Paez AG, Parks BG, Schon LC, Hasenboehler EA (2016). Early weightbearing after operatively treated ankle fractures: a biomechanical analysis. Foot Ankle Int.

[17] Lorente A, Palacios P, Lorente R, Mariscal G, Barrios C, Gandía A (2019). Orthopedic treatment and early weight-bearing for bimalleolar ankle fractures in elderly patients: quality of life and complications. Injury.

[18] Cuschieri S (2019). The STROBE guidelines. Saudi J Anaesth.

[19] Weber BG (1972). Die Verletzungen des oberen Sprunggelenkes. 2nd ed.

[20] Lauge-Hansen N (1952). Fractures of the ankle IV. Clinical use of genetic roentgen diagnosis and genetic reduction. Arch Surg.

[21] Gandek B, Ware JE, Aaronson NK, Apolone G, Bjorner JB, Brazier JE (1998). Crossvalidation of item selection and scoring for the SF-12 health survey in nine countries: results from the IQOLA project. Int Qual Life Assess J Clin Epidemiol.

[22] Mahoney FI, Barthel D (1965). Functional evaluation: the Barthel index. Md State Med J.

[23] Mayoral AP, Ibarz E, Gracia L (2019). The use of Barthel index for the assessment of the functional recovery after osteoporotic hip fracture: one year follow-up. PLoS One.

[24] Hulsbæk S, Larsen RF, Rosthøj S (2018). The Barthel index and the cumulated ambulation score are superior to the de Morton mobility index for the early assessment of outcome in patients with a hip fracture admitted to an acute geriatric ward. Disabil Rehabil.

[25] Kammerlander C, Riedmüller P, Gosch M (2012). Functional outcome and mortality in geriatric distal femoral fractures. Injury.

[26] Michelson JD, Magid D, McHale K (2007). Clinical utility of a stability-based ankle fracture classification system. J Orthop Trauma.

[27] Sanders DW, Tieszer C, Corbett B (2012). Canadian orthopedic trauma society: operative versus nonoperative treatment of unstable lateral malleolar fractures: a randomized multicenter trial. J Orthop Trauma.

[28] Busija L, Pausenberger E, Haines TP, Haymes S, Buchbinder R, Osborne RH (2011). Adult measures of general health and health-related quality of life: Medical Outcomes Study Short Form 36-Item (SF-36) and Short Form 12-Item (SF-12) Health Surveys, Nottingham Health Profile (NHP), Sickness Impact Profile (SIP), Medical Outcomes Study Short Form 6D (SF-6D), Health Utilities Index Mark 3 (HUI3), Quality of Well-Being Scale (QWB), and Assessment of Quality of Life (AQoL). Arthritis Care Res.

[29] Andersson G, Surgeons AAoO. United States Bone and Joint Initiative: The Burden of Musculoskeletal Diseases in the United States (BMUS). Rosemont, IL; 2014

[30] Nilsson G, Nyberg P, Ekdahl C, Eneroth M (2003). Performance after surgical treatment of patients with ankle fractures–14- month follow-up. Physiother Res Int.

[31] Hancock MJ, Herbert RD, Stewart M (2005). Prediction of outcome after ankle fracture. J Orthop Sports Phys Ther.

[32] Lin C-WC, Moseley AM, Herbert RD, Refshauge KM (2009). Pain and dorsiflexion range of motion predict short- and medium-term activity limitation in people receiving physiotherapy intervention after ankle fracture: an observational study. Aust J Physiother.

[33] Berg HE, Larsson L, Tesch PA (1997). Lower limb skeletal muscle function after 6 wk of bed rest. J Appl Physiol.

[34] Hather BM, Adams GR, Tesh PA, Dudley GA (1992). Skeletal muscle responses to lower limb suspensions in humans. J Appl Physiol.

[35] LeBlanc A, Gogia P, Schneider V, Krebs J, Schonfeld E, Evans H (1988). Calf muscle area and strength changes after five weeks of horizontal bed rest. Am J Sports Med.

[36] Davies CTM, Rutherford IC, Thomas DO (1987). Electrically evoked contractions of the triceps surae during and following 21 days of voluntary leg immobilization. Eur J Appl Physiol.

[37] Jones KB, Maiers-Yelden KA, Marsh JL, Zimmerman MB, Estin M, Saltzman CL (2005). Ankle fractures in patients with diabetes mellitus. J Bone Joint Surg (Br).

[38] Costigan W, Thordarson DB, Debnath UK (2007). Operative management of ankle fractures in patients with diabetes mellitus. Foot Ankle Int.

[39] Flynn JM, Rodriguez-del Rio F, Piza PA (2000). Closed ankle fractures in the diabetic patient. Foot Ankle Int.

[40] Jupiter DC, Humphers JM, Shibuya N (2014). Trends in postoperative infection rates and their relationship to glycosylated hemoglobin levels in diabetic patients undergoing foot and ankle surgery. J Foot Ankle Surg.

[41] Nasell H, Ottosson C, Tornqvist H, Linde J, Ponzer S (2011). The impact of smoking on complications after operatively treated ankle fracturesda follow-up study of 906 patients. J Orthop Trauma.

[42] Kim JW, Byun SE, Chang JS (2014). The clinical outcomes of early internal fixation for undisplaced femoral neck fractures and early full weight-bearing in elderly patients. Arch Orthop Trauma Surg.

[43] Bazarov I, Peace RA, Lagaay PM, Patel SB, Lyon LL, Schuberth JM (2017). Early protected Weightbearing after ankle fractures in patients with diabetes mellitus. J Foot Ankle Surg.

